# Experimental assessment of permethrin-fipronil combination in preventing *Leishmania infantum* transmission to dogs under natural exposures

**DOI:** 10.1016/j.vpoa.2020.100026

**Published:** 2020-05-18

**Authors:** Elias Papadopoulos, Athanasios Angelou, Maxime Madder, Wilfried Lebon, Frederic Beugnet

**Affiliations:** a Laboratory of Parasitology and Parasitic Diseases, School of Veterinary Medicine, Aristotle University of Thessaloniki, Greece; b Clinglobal, The Tamarin Commercial Hub, Jacaranda Avenue, Tamarin, 90903, Mauritius; c Boehringer-Ingelheim Animal Health, 29 Av Tony Garnier, 69007, Lyon, France

**Keywords:** Controlled study, *Leishmania infantum*, Permethrin, Prevention, Topical insecticide

## Abstract

Canine vector-borne disease transmission can be reduced with regular use of repellent insecticides. The objective of this year-long experimental study was to assess the efficacy of a topical formulation of fipronil/permethrin (Frontline Tri-Act®) in preventing transmission of *Leishmania infantum* by sandflies. This clinical field trial was conducted in Xanthi (Northern Greece), an area highly endemic for canine leishmaniosis, from April 2018 to March 2019. Forty purpose-bred Beagle dogs, testing negative for *L. infantum* prior to study initiation, were enrolled in the study, which consisted of three phases: Phase 1 (field exposure phase) took place from Day 0–196. The dogs were randomly allocated to two groups, group 1 (sham-treated topically with sterile water) and group 2 (treated topically with Frontline Tri-Act®). Dogs in both groups were housed in two subunits of an open-air kennel for a period of 7 months, spanning the *Leishmania* transmission season. All dogs were treated or sham-treated on Days 0, 28, 56, 84, 112, 140 and 168. Clinical examinations, PCR analysis of conjunctival swabs, and serological tests were performed on a monthly basis. Sandflies were collected every 2 weeks, during a 12 -h period overnight using light traps. Each collection was placed in a container and kept refrigerated until speciation and PCR analysis could be performed. In the second phase of the study, from Day 197 to 252, the dogs were moved into a protected environment (insect-proof protected environment phase). CDC light traps were activated every 2 weeks inside and outside the kennels to ensure the vector-free status of the facility. Monthly clinical examinations, including PCR analysis of conjunctival swabs, and serological tests continued. At the end of the phase 2, bone marrow samples were collected on all dogs. Phase 3 (the final post-winter check) took place from Day 253 to 350. Dogs were released and adopted by individual private owners on Day 253. Follow up analyses included blood collection for SNAP tests and conjunctival swaps for PCR analysis on Days 304 and 350. Additionally, bone marrow collections were also performed on Day 350.

Presence of sandflies was observed only in the phase 1 exposure period, and 1714 sandflies were collected (1427 females and 287 males). Two species were identified, *Phlebotomus perniciosus* var*. tobbi* and *Phlebotomus neglectus*. Out of the 62 pooled samples of sandflies assessed by PCR, three were considered positive (4.8 %). By the end of the study, 35 % of the Group 1 dogs (7/20) became positive based on PCR (conjunctival swab and bone marrow) and 30 % (6/20) based on SNAP/IFAT and ELISA tests, while all the dogs in the Frontline Tri-Act® treated group 2 remained negative for all tests (G1 vs G2, p = 0.008). All tests identified the same positive animals, and PCR allowed the detection of one additional infected dog. This clinical field trial demonstrated that monthly administration of Frontline Tri-Act® to dogs exposed to *Leishsmania* infection in a high endemic area provided 100 % preventive efficacy against transmission of *L. infantum*.

## Highlights

• Controlled experimental field study allowing natural exposure to sandflies.
• Two groups of 20 dogs each in two close sub-unit kennels.
• 7/20 dogs infected by *L. infantum* in the untreated group.
• 0/20 dogs infected by *L. infantum* in the fipronil-permethrin monthly treated group.
• 100 % protection obtained during this one-year study.

## Introduction

Canine leishmaniosis (CanL) is an infectious disease caused by the proliferation of the protozoan flagellate parasite *Leishmania infantum* in cells of the reticulo-endothelial system. This parasite is mainly transmitted by the bite of phlebotomine sandflies (*Phlebotomus* in the Old World, *i.e.* Africa, Asia, Europe; and *Lutzomyia* in the Americas) (Killick-Kendrick, 1979, Killick-Kendrick, 1999). While dogs constitute the main reservoir, *L. infantum* can also infect other mammals like lagomorphs, rodents, foxes, cats, horses, and humans.

CanL is highly endemic in countries around the Mediterranean basin, but also Northern Africa, Southern Asia, and Central and South America (Palatnik-de-Soussa and Day, 2011). In endemic countries, the seroprevalence in dogs can vary from less than 5 % to more than 50 % (Palatnik-de-Soussa and Day, 2011, Maia and Cardoso, 2015). But in a single country, the distribution can be very heterogeneous between endemic foci (with high seroprevalence, multiple clinical cases, active and infected vectors) and ectopic or new foci (with low prevalence, few cases and no or very few vectors but a possible non-vectorial transmission) (Grammicia, 2011, EFSA Panel Animal Health and Welfare, 2015, Mendoza-Roldan et al., 2020).

Besides endemic areas, recent surveys have demonstrated a gradual spread to what were previously considered non-endemic areas (Grammicia, 2011, Beugnet and Chalvet-Monfray, 2013, Maia and Cardoso, 2015). In fact several studies have described new outbreaks from Southern or Central Italy to Northern Italy, such as Tuscany, Marche and Emilia Romagna (Mendoza-Roldan et al., 2020); a spread to the West and Northwest of France; and CanL is now becoming endemic in Balkans, Romania with extension towards Central and Northern Europe (Colella et al., 2019). Diagnosis of infection is not rare in Northern Europe, as infections commonly occur in dogs that have travelled to the South.

CanL is a major concern in veterinary medicine and public health for several reasons (Grammicia, 2011, Otranto and Dantas-Torres, 2013, Miró et al., 2017): (i) the prevalence of CanL in endemic areas has been increasing, in addition these geographies are popular tourist destinations. As a result, the number of reported clinical cases in non-endemic countries and/or areas has also increased by importing or travelling with apparently healthy but infected dogs returning to non-endemic areas; (ii) the non-vectorial transmission (sexual, vertical, blood exchange) is responsible for new foci in previously non-endemic countries, such as the United States of America, or even in dogs that have never been in endemic areas; and (iii) CanL is a potentially fatal disease. CanL is a zoonosis and human cases have been reported in endemic areas where the prevalence of CanL in dogs is high.

Female sandflies are the only arthropods capable of biological transmission of *Leishmania* promastigotes to dogs under natural conditions (Killick-Kendrick, 1999). *Phlebotomus perniciosus* is one of the major vectors of canine leishmaniosis in Southern Europe. Depending the taxonomy, many species are described, and sometimes regrouped as sub-species of a complex species. This is a case for *Phlebotomus (Larroussius) tobbi*, which has been considered as a variety or sub-species of *P. perniciosus* (Simir, 1932, Killick-Kendrick et al., 1991). Other *Phlebotomus* spp. are also involved as vectors in North Africa, South-eastern Europe and Central Asia, e.g. *Phlebotomus ariasi, P. perfiliewi,* and *P. sergenti*. Over the past decades, there has been an increase in sandfly geographical distribution and density, which can be attributed to climatic and ecological changes. Thus, CanL is spreading northwards in Europe with some examples such as the French Pyrenees, the Toulouse region, and the Rhône valley in southern France, as well as Catalonia in northeastern Spain, and Galicia in northern Spain (Grammicia, 2011, Maia and Cardoso, 2015, Miró et al., 2017).

There are two essential strategies to limit the transmission of *L. infantum* to dogs and humans (Dantas-Torres and Otranto, 2016, Miró et al., 2017, Otranto and Dantas-Torres, 2013): (i) the control of the canine reservoir by using insecticides with repellent activity to prevent sandfly bites, by treating infected dogs, and by vaccinating dogs in enzootic areas; and (ii) the control and reduction of the vector density by targetting the sandfly breeding ecosystems. Depending on the climate, the transmission occurs when vectors are active. Mainly from spring to autumn in Europe (March/April to October/November). However, the strategy of culling dogs, that is still in place in some countries or states, especially in Brazil, has no demonstrable benefit (Dantas-Torres et al., 2019).

Repellent insecticides, most of which belong to the pyrethroid family, provide efficacy that has been demonstrated both experimentally and in field studies (deltamethrin or permethrin-impregnated collars and spot-ons are available) (Beugnet and Franc, 2012). Collars containing deltamethrin or flumethrin, and spot on containing permethrin have proven to decrease the prevalence of CanL in endemic areas and also decreases the risk of transmission to humans (Brianti et al., 2016, Foglia Manzillo et al., 2006, Giffoni et al., 2002, Maroli et al., 2001, Otranto et al., 2013, Otranto et al., 2010, Otranto et al., 2007).

Frontline Tri-Act® is a topical ectoparasiticide for dogs which contains fipronil (6.01 % w/v) and permethrin (44.88 % w/v) as active ingredients.

Frontline Tri-Act® is indicated for the treatment and prevention of fleas and ticks but also repels and kills flying insects such as stable flies, mosquitoes and sandflies (Dumont et al., 2015a, Dumont et al., 2015b, Dumont et al., 2015c, Fankhauser et al., 2015a, Fankhauser et al., 2015b,). The insecticidal and repellent effect against *Phlebotomus* spp. has been demonstrated in experimental studies (Dumont et al., 2015c). A repellent effect >90 % of Frontline Tri-Act® has been observed against sandflies (*P. perniciosus*) for three weeks (through an anti-feeding activity) and maintains >80 % for a month.

Based on the repellent activity of Frontline Tri-Act® against sandflies, it can be hypothesized that regular applications would reduce the number of sandfly bites, therefore indirectly reducing potential *Leishmania* transmission and clinical leishmaniosis.

A similar hypothesis was proposed on tick repellent action and the potential to reduce the risk of tick borne pathogen transmission. A clear reduction of the risk was demonstrated in transmission studies of *Babesia canis* and of *Ehrlichia canis* and have been published (Beugnet et al., 2014, Jongejan et al., 2015).

The efficacy of Frontline Tri-Act in preventing leishmaniosis in dogs was preliminarily assessed in a six month experimental study. The study was a positive controlled (deltamethrin collar) study, with five monthly treatments of fipronil-permethrin were given from May to October 2015 (Papadopoulos et al., 2017). In this study conducted in Xanthi, in Greece, where a high seroprevalence of leishmaniosis (48 %) was known from previous epidemiological surveys (Gallidis et al., 2016, unpublished data), 3/25 dogs were seropositive to *Leishmania* in the deltamehrin treated Group versus 0/31 dog in the fipronil-permethrin treated Group. In another six month field study conducted in Sicily, following 41 dogs treated monthly with both Frontline Tri-Act and NexGard Spectra (afoxolaner + milbemycin). Six of those 41 dogs were positive at the beginning of the study, and the 35 *Leishmania* negative dogs remained negative until the end (Abbate et al., 2018).

In the present year-long study, conducted as a single centre field study using natural exposure, we examined the ability of an insecticidal-repellent formulation to prevent *Leishmania* infection in dogs.

## Materials and methods

This was a negative controlled field efficacy study, with blinding using a randomized block design, conducted between April 2018 and March 2019 in order to include the seasonal transmission of *Leishmania* by sandflies (spring to autumn) and to allow sufficient time for potential seroconversion. The animals and the facility were set-up as an experimental study, but performed in the field to allow natural challenge. The study was designed in compliance with Good Clinical Practices as described in the International Cooperation on Harmonization of Technical Requirements for Registration of Veterinary Medicinal Products, VICH Guideline 9. The study was approved by both the Boehringer-Ingelheim Animal Health and the Veterinary Faculty of Thessaloniki ethical committees.

This study was conducted in Sounio, Xanthi region (north-eastern Greece, 41°07′37.6″N 25°02′56.6″E) where there is a high seroprevalence of leishmaniosis (previously published as being around 48 %) (Boutsini et al., 2018; Gallidis et al., 2016; Ntais et al., 2013).

The dogs used were laboratory purpose-bred Beagle dogs, serologically and PCR-negative for *Leishmania.* A negative control group was included in the study. The facility, a new dog kennel, specifically built for the study in the countryside as an open kennel, included two separated and identical subunits (See supplementary pictures). The two units were separated 20 m apart, and face-to-face, in order to be exposed to a similar sandfly density.

### Inclusion criteria and allocation

Dogs were allocated to study groups if they met the following criteria: (i) clinically healthy; (ii) females not pregnant; (iii) PCR negative and seronegative for L. *infantum* infection at the initiation of the study; and (iv) not excessively aggressive in that they would pose a danger to study site personnel.

Forty Beagle dogs (30 males and 10 females), weighing 9.0–15.5 kg on Day 0, all tested negative for the presence of *L. infantum* infection (by enzyme-linked immunosorbent assay (ELISA) on blood samples and polymerase chain reaction (PCR) on both conjunctival swaps and bone marrow), and were randomly allocated to two study groups, group 1 (sham-treated with sterile water) and group 2 (treated with Frontline Tri-Act®). The decision to include 40 dogs was related to the expected exposure, if at least 5 infections in the control group would lead to a significant difference (i.e. p = 0.047 Fisher exact’s test if 5/20 dogs were infected in the negative control and 0/20 in the treated group).

### Study design

The study was conducted in three successive phases:

#### Phase 1 (field exposure phase): Day 0–196 (April - October 2018)

Prior the start of the study, blood samples were collected from all 40 included dogs for haematology and clinical chemistry, and a urine sample was collected from each dog for urinalysis.

Over a period of seven months covering the *Leishmania* transmission season, all dogs were treated topically monthly with either sterile water and served as negative controls or with the investigational veterinary product (IVP) to evaluate preventive efficacy, depending on their group allocation (Days 0, 28, 56, 84, 112, 140 and 168). The volume of IVP/sterile water administered was calculated according to the dogs’ individual body weight.

Clinical examinations were conducted monthly, with all dogs weighed prior to dosing. PCR analysis of conjunctival swabs, and serological SNAP tests were also performed on a monthly basis to determine the presence of *L. infantum* infection in the dogs.

Dogs in both groups were continuously and similarly challenged by the presence of naturally occurring sandflies. To confirm the presence of the vectors, sandflies were trapped using Center for Disease Control (CDC) light traps once every two weeks, for a 12 h period overnight, collected and then kept refrigerated until examination. Sandfly examinations included counting, morphological identifying up to species level and molecular analysis for the presence of *Leishmania* spp.

#### Phase 2 (Insect-proof protected environment phase): Day 197–252 (October - December 2018)

Phase 2 started at the end of autumn and continued through winter, after confirming the end of the sandfly season. Throughout phase 2, the original kennel units were covered with insect-proof fine-screen nets, which would not allow sandflies to go through (See supplementary pictures). The net was made with a commercial insect proof-gauze from Ixtiaroglou textile (13 Ermou street. Thessaloniki), impregnated in a solution of deltamethrin. The outside area of the kennel and the nets were also sprayed with deltamethrin after installment. CDC light traps were activated every 2 weeks inside and outside the two kennels to check for the presence of active vectors and possible failures of insect-proofing. In addition, all dogs were also fitted with deltamethrin collars in order to decrease the risk of any arthropod bites.

As in Phase 1, clinical examinations and weighing of all dogs were conducted monthly. Conjunctival swab sampling for PCR analysis, and serology SNAP tests were performed on a monthly basis to ensure that new circulating *L. infantum* parasites or late seroconversions would be recorded.

At the end of the phase 2, the following were collected: bone marrow extract for PCR analysis; blood samples to do the three serological tests (SNAP, ELISA and IFAT) and haematology and clinical chemistry; and urine for urinalysis.

#### Phase 3 (Final post-winter check) Day 253–350 (December - March 2019)

Phase 3 occurred from winter to early spring. At the end of phase 2, the dogs were adopted out to individual private owners, so housing and management were as per owners’ preference. Blood samples for SNAP, ELISA and IFAT, and conjunctival swabs for PCR analysis were collected on Days 304 and 350. Bone marrow collections were also conducted for PCR analysis on all dogs remaining in the study on Day 350. One dog with ID 20 in group 2 was struck and killed by an automobile, as recorded on Day 296.

### Molecular tests for L. *infantum* (DNA extraction and PCR)

PCR was performed as described by de Almeida Ferreira et al., 2008 and Pilatti et al., 2009.

DNA from conjunctival swabs was extracted using DNeasy Blood and Tissue kit (Qiagen, Germany) following the manufacturer’s instructions. Purified DNA was eluted in elution buffer.

Bone marrow aspirates (500 μL) were incubated on ice for 20 min using 2.5 mL of lysis buffer. After centrifugation (3000 *g*, 10 min), cell pellets were incubated with 150 μL of lysis buffer and 5 μL of proteinase K (56 °C, 12 h). Extraction was performed by adding 125 μL of phenol–chloroform–isoamylalcohol to 150 μL of lysate. Organic and aqueous phase were separated by centrifugation at 15,000*g* for 2 min. For DNA precipitation, 50 μL of isopropanol and 50 μL of 4 M ammonium acetate (pH 7.5) were added to the supernatant. After centrifugation at 15,000 *g* for 5 min, the precipitate was washed using 70 % ethanol. The DNA pellet was resuspended in 50 μL of buffer (Ferrer et al., 2006; Geisweid et al., 2013).

Genomic DNA was extracted from individual insects using DNeasy Blood & Tissue Kit (Qiagen, GmbH, Hilden, Germany) in accordance to the manufacturer’s instructions.

For the molecular analysis, *L. infantum* specific oligonucleotide primers N13A (5′-AACTTTTCTGGTCCTCCGGG-3′) and N13B (5′-CCCCCAGTTTCCCGCCC-3′) were selected to amplify a 120-base-pair fragment of the *Leishmania* kinetoplast DNA minicircle (Pilatti et al., 2009). To ensure that negative results corresponded to true negative samples rather than a false negative result, one sample DNA was also amplified for β-actin by using a forward primer (5′-GACAGGATGCAGAAGGAGAT-3′) and a reverse primer (5′-TTGCTGATCCACATCTGCTG-3′) at 0.3 μM of each primer in the same conditions. Amplified fragments were analyzed by electrophoresis in a 3 % agarose gel containing ethidium bromide (0.5 μg/ml) (Francino et al., 2006).

### Serology

#### ELISA

The ELISA was performed as described by Athanasiou et al., 2014. The ELISA used water-soluble proteins of promastigote forms of L. *infantum* (zymodeme MON1) as antigens and goat anti-dog IgG antibodies (gamma heavy chain specific) conjugated to alkaline phosphatase (Kirkegaard & Perry Laboratories, Inc., Gaithersburg, MD) as detection antibodies. The cut‐off value was determined by adding three standard deviations to the average absorbance at 405 nm of the negative control sera.

#### IFAT

The IFAT was performed as described by Miro et al., 2009 and Athanasiou et al., 2014. For IFAT, commercially available slides coated with *L. infantum* promastigotes (Fluoleish, BVT, France) and conjugate (anti-Dog IgG, Sigma) were used). A titre greater or equal to the cut-off of 1/80 was considered positive (The range was 1/40, 1/80, 1/400 and 1/800).

#### SNAP®-Leishmania

Commercial anti-*Leishmania* antibody SNAP tests from IDEXX were used on a monthly basis.

### Sandflies identification

All specimens were individually identified using morphological keys as described by Killick-Kendrick et al., 1991 and Dantas-Torres et al., 2014.

### Statistical analysis

The number of positive and negative dogs in each group were compared by using a Fisher exact test. The percentage of efficacy, i.e. the % of protection was calculated by using the Abbot’s formula as followed: % Protection = [((number of infected dogs in Group 1) – (number of infected dogs in Group 2))/ (number of infected dogs in Group 1)]* 100.

## Results

### Confirmation of the sandfly challenge during phase 1

The presence of sandflies was documented by CDC light trap collections only during the exposure phase 1 (from Day 28 to Day 182) (Table 1). The light trap collections, located inside and outside the net in phase 2, were all negative, thus confirming the absence of exposure during the phase 2 period.

**Table 1:** Sandfly counts during phase 1 and PCR on pooled female sandflies.

Phase 1 - Sandfly collection	Females	Males	Total	number of PCRs (pools of females)	PCR Results on pooled sandflies
**Day 28 (Visit 3)**	8 *P. perniciosus*	0	8	2 pooled sample (n = 3 and 5)	negative
**Day 42 (Visit 4)**	13 *P. perniciosus*	1 *P. perniciosus*	14	2 pooled (n = 5)	negative
**Day 56 (Visit 5)**	11 *P. perniciosus*	1 *P. perniciosus*	12	2 pooled (n = 5 and 6)	negative
**Day 70 (Visit 6)**	67 *P. perniciosus*	18 *P. perniciosus*	85	3 pooled (n = 15 each)	negative
**Day 84 (Visit 7)**	169 *P. perniciosus*	13 *P. perniciosus*	182	6 pooled (n = 15 each)	1 positive pool (faint band)
**Day 98 (Visit 8)**	177 *P. perniciosus*	20 *P. perniciosus*	197	6 pooled (n = 15 each)	negative
**Day 112 (Visit 9)**	170 *P. perniciosus*	19 *P. perniciosus*	189	6 pooled (n = 15 each)	negative
**Day 126 (Visit 10)**	186 *P. perniciosus*	32 *P. perniciosus*	218	7 pooled (n = 15 each)	2 positive pool (faint band)
**Day 140 (Visit 11)**	114 *P. perniciosus*	28 *P. perniciosus*	142	5 pooled (n = 15 each)	negative
	92 *P. neglectus*	19 *P. neglectus*	111	4 pooled (n = 15 each)	negative
**Day 154 (Visit 12)**	152 *P. perniciosus*	50 *P. perniciosus*	202	6 pooled (n = 15 each)	negative
	21 *P. neglectus*	5 *P. perniciosus*	26	1 pooled (n = 15)	negative
**Day 168 (Visit 13)**	139 *P. perniciosus*	47 *P. perniciosus*	186	6 pooled (n = 15 each)	negative
	33 *P. neglectus*	12 *P. neglectus*	45	2 pooled (n = 15 each)	negative
**Day 182 (Visit 14)**	75 *P. perniciosus*	22 *P. perniciosus*	97	4 pooled (n = 15 each)	negative
**Total**	**1427 (83.30 %)**	**287 (16.69 %)**	**1714**	**62 pools (872 sandflies)**	**3 positive (4.8 % of pools)**

Two sandfly species were identified during phase 1, *Phlebotomus perniciosus* var*. tobbi* and *Phlebotomus neglectus*. In total, 1427 females sandflies were trapped (among them, 1281 were *P. perniciosus* sensu lato sandflies and 146 were *P. neglectus*) (Table 1). Females represented around 83.3 % of the trapped sandflies. A total of 62 pools of female sandflies (3–15 females per pool), including 872 females, were assessed. Three PCR results gave a positive faint band (4.8 %), which could be interpreted as a small amount of amplified sequences of *Leishmania infantum*. Faint bands are usually observed in case of low DNA level for several reason like the number of amplification cycles, the temperature, the number of sandflies per pooled sample, or the choice of primers.

### Detection of *Leishmania* infection

#### Serological results

Prior to the study start, the ELISA, IFAT and SNAP results were negative for all dogs.

At the end of phase 2 (Day 252), four animals in group 1 (Animal IDs 6, 23, 33 and 37) had positive ELISA, IFAT and SNAP results, whereas no positive was found in group 2 dogs (Table 2).

**Table 2:** Serological (ELISA, IFAT, SNAP tests) and PCR (conjunctival swabs and bone marrow aspirates) follow-up of dogs.

Days	Group 1 Untreated control		Group 2 Frontline Tri-Act treated
	Positive n / N (%), Dog ID, Technique		
	Serology	PCR	Serology and PCR
Day 112	1/20 (5 %)	0/20 (0 %)	0 / 20 (0 %)
	6	PCR conjunctival swabs	ELISA, PCR conjunctival swabs
	ELISA		
Day 140	2/20 (10 %)	2/20 (10 %)	0 / 20 (0 %)
	6, 23	6, 37	SNAP, PCR conjunctival swabs
	SNAP	PCR conjunctival swabs	
Day 168	3/20 (15 %)	4/20 (10 %)	0 / 20 (0 %)
	6, 23, 33	6, 23, 33, 37	SNAP, PCR conjunctival swabs
	SNAP	PCR conjunctival swabs	
Day 252	4/20 (10 %)	4/20 (10 %)	0 / 20 (0 %)
	6, 23, 33, 37	6, 23, 33, 37	ELISA, SNAP, IFAT, PCR conjunctival swabs & bone marrows
	ELISA, SNAP, IFAT	PCR conjunctival swabs & bone marrows	
Day 350	6 / 20 (20 %)	7 / 20 (20 %)	0 / 20 (0 %)
	3, 6, 23, 30, 33, 37	3, 6, 15, 23, 30, 33, 37	ELISA, SNAP, IFAT, PCR conjunctival swabs & bone marrows
	ELISA, SNAP, IFAT	PCR conjunctival swabs & bone marrows	
	p = 0.02 (Fischer’s exact test compared to treated group)	p = 0.0083(Fischer’s exact test compared to treated group)	

Group 1: Dogs were treated with sterile water, intended as a "sham-treatment" to maintain blinding, as a topical spot-on on Days 0, 28, 56, 84, 112, 140 and 168 Group 2: Dogs were treated with Frontline Tri-Act® as a topical spot-on on Days 0, 28, 56, 84, 112, 140 and 168.

At the end of phase 3 (Day 350), two more animals in group 1 (Animal IDs 3 and 30) became positive with ELISA, IFAT and SNAP, leading to six animals that were positive on serological results in group 1 (Table 2). All dogs in group 2 remained negative by serology.

The difference between the two groups was statistically significant by the end of the study on Day 350 with a p = 0.02 (Fisher's exact p-values) for all serological methods, with six seropositive dogs in the control group and zero in the treated group. The serological tests were all concordant with the diagnosis of the same six dogs, with four dogs diagnosed at the end of phase 2, and two additional at the end of phase 3.

#### PCR tests

Prior to study start, PCR analysis were negative for all dogs.

Seven dogs in group 1 tested positive for *L. infantum* infection on PCR results from conjunctival swabs and/or bone marrow aspirations during the study. Animal ID 6 and Animal ID 37 were positive from Day 140 onwards and animal ID 23 and animal ID 33 were positive from Day 168. Animals IDs 3, 15 and 30 became positive on Day 350. All animals in group 2 remained negative on PCR results from conjunctival swabs and bone marrow throughout the study (Table 2).

The two PCR tests were concordant in their results. As for serology, some dogs were diagnosed at a late stage (phase 3), indicating that the incubation can be longer than 9 months. PCR tests allowed identification of one additional infected dog (ID 15), that was serologically negative at Day 350.

#### Clinical leishmaniosis

All animals presented with normal haematology and clinical chemistry values on all monthly assessments prior to Day 350. At the Day 350 assessment, four *Leishmania* infected dogs in group 1 (IDs 6, 23, 33 and 37) presented with abnormalities in renal function determined by urine protein⁄creatinine ratio analysis. The presence of renal lesions related to immune-complexes is well described in the pathogenesis of leishmaniosis, however no biopsies were performed. None of the dogs presented with a decline in general condition (amyotrophy and weight loss), nor were skin lesions indicative of canine leishmaniosis observed. The clinical follow-up confirmed that leishmaniosis is a chronic disease with a very low development of clinical signs.

## Discussion

The two groups showed significantly different results, which can be attributed only to the repellent treatment as we can consider that the exposure to sandflies was the same in both subunits.

The two kennel subunits were facing each other and were separated by 20 m of land. Both were close to vegetation and stones, facing the same risk of exposure to sandflies which are known to fly around 500−2000 m from their birthplace (Killick-Kendrick, 1999).

In the present clinical field study, monthly administrations of a combination of fipronil and permethrin (Frontline Tri-Act®) provided a complete preventive efficacy (100 %) against canine leishmaniosis due to *L. infantum* in a highly endemic area with an observed incidence of 30–35 % (Ntais et al., 2013). These results are in accordance with other field studies following the impact of repellent treatment on dogs in endemic areas (Maroli et al., 2001, Giffoni et al., 2002, Foglia Manzillo et al., 2006, Otranto et al., 2010, Otranto et al., 2007, Otranto et al., 2013, Brianti et al., 2016).

A preliminary field study had obtained similar results, i.e. no infected dogs in the fipronil-permethrin monthly treated dogs, but there was no negative control group to confirm the exposure (Papadopoulos et al., 2017). Nevertheless, exposure was present, as 4 dogs from the positive control group (deltamethrin collar) were infected in this study.

In the present study a negative control group was included, and 7 dogs out of 20 controls were infected. Both serological tests (3 different*, i.e.* ELISA, IFAT and SNAP) and molecular PCR tests (2 different sampling techniques, i.e. conjunctival swab and bone marrow aspiration) were conducted. All tests were highly concordant with only one dog being PCR positive and serologically negative. Based on previous publication indicating that it may take time for infected dogs to become positive, it was decided to continue to follow the dogs for six months after the transmission season (Solano-Gallego et al., 2011).

In the present study, the collection of sandflies provided additional evidence for the exposure to *L. infantum*, even though only three pools of female sandflies provided a faint band that was considered as positive by the authors. Considering that one female was positive in each pool, it gives an estimate of infection of 0.34 % which is very similar to previous publication (Boutsini et al., 2018). In this previous study also conducted in Greece (Boutsini et al., 2018), out of 3254 sandflies trapped, 1448 (44.43 %) were female and 241 (16.64 %) of the females were blood fed while *L. infantum* DNA was detected in 0.41 % of them.

Only one site was studied due to the cost, the logistic and the availability of trained personnel to work on canine leishmaniosis. For ethical reasons, a minimum number of dogs was included.

The regular use of formulations containing pyrethroids on dogs is advised to decrease the risk of *Leishmania* infection by several scientific groups like Leishvet, in white papers like EFSA scientific opinion on canine leishmaniosis and in scientific reviews (Otranto and Dantas-Torres, 2013; Dantas-Torres et al., 2014; EFSA, 2015; Miró et al., 2017; Otranto, 2018). It is also recommended to maintain the repellent protection in combination with anti-*Leishmania* vaccine.

Based on the repellent and insecticidal activity of the combination fipronil-permethrin, on the preliminary field study, and on the present field study with an experimental design including a negative control group, we can conclude that the monthly use of the fipronil-permethrin combination (Frontline Tri-Act®) indirectly reduces the risk of *Leishmania* transmission by sandflies.

## Declaration of Competing Interest

This clinical study was funded by Boehringer Ingelheim Animal Health, 29 Avenue Tony Garnier, 69007, Lyon of which Frédéric Beugnet and Wilfried Lebon are employees.

All authors voluntarily publish this article and have no personal interest in these studies other than publishing the scientific findings that they have been involved in via planning, initiating, monitoring and conducting the investigations and analysing the results.

## Disclaimer

Frontline Tri-Act/Frontect® is a registered trademark of Boehringer Ingelheim Animal Health.

All other brands are the property of their respective owners.

This document is provided for scientific purposes only. Any reference to a brand or a trademark herein is for informational purposes only and is not intended for a commercial purpose or to dilute the rights of the respective owner(s) of the brand(s) or trademark(s).

## CRediT authorship contribution statement

**Elias Papadopoulos:** Conceptualization, Investigation, Methodology, Validation. **Athanasios Angelou:** Investigation. **Maxime Madder:** Data curation, Formal analysis, Investigation, Methodology, Supervision, Validation. **Wilfried Lebon:** Funding acquisition, Project administration, Supervision. **Frederic Beugnet:** Conceptualization, Formal analysis, Funding acquisition, Methodology, Supervision, Validation, Visualization, Writing - original draft, Writing - review & editing.
