# Supplementary material for: Experimental assessment of permethrin-fipronil combination in preventing Leishmania infantum transmission to dogs under natural exposures
Source: Vet Parasitol X. 2020 May 18;3:100026. doi: 10.1016/j.vpoa.2020.100026 (PMC7458379; doi:10.1016/j.vpoa.2020.100026)

**Picture 1**: **View of the two newly built kennel subunits in countryside for the study**


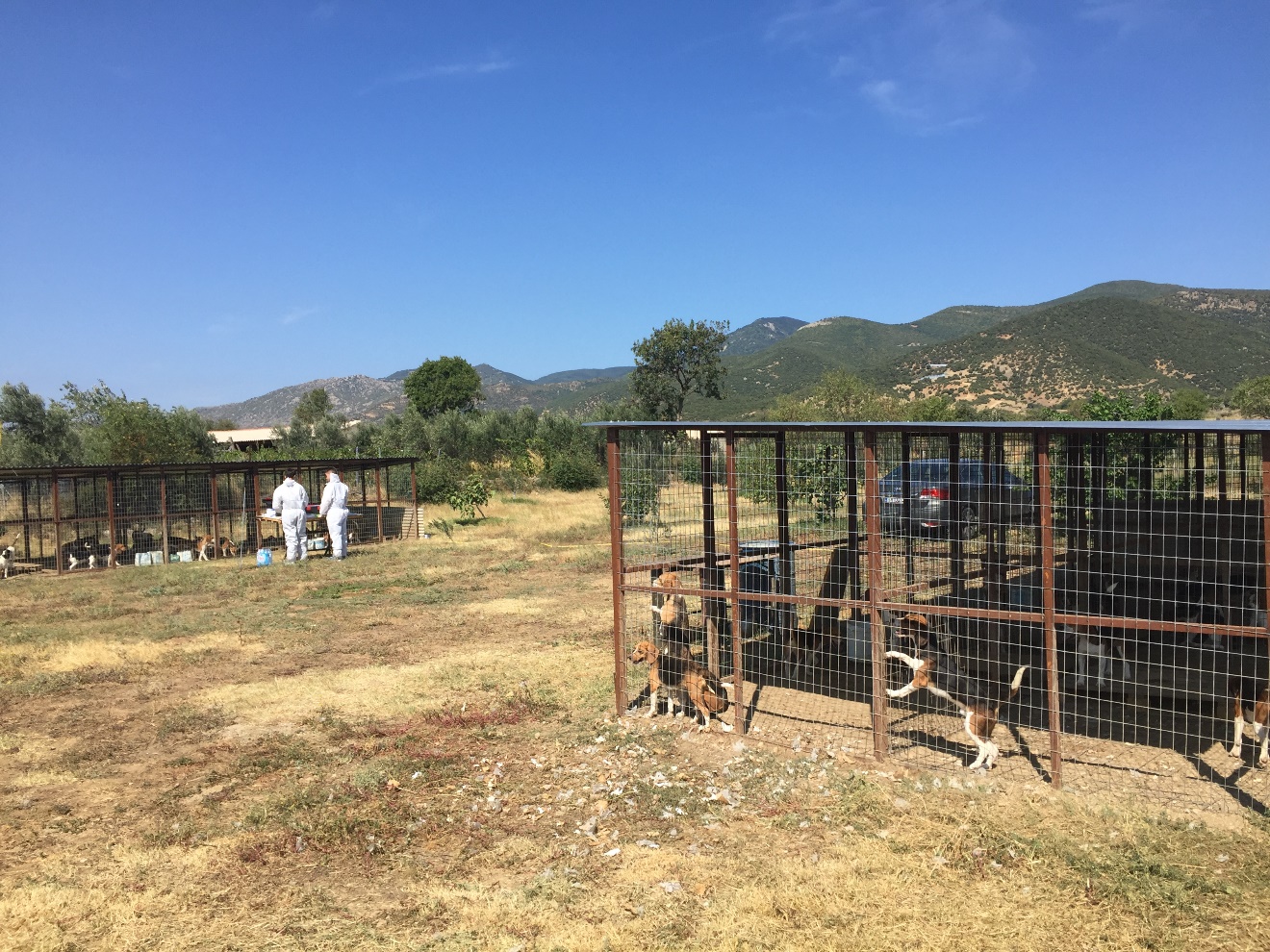


**Picture 2: Untreated Group 1 subunit kennel**


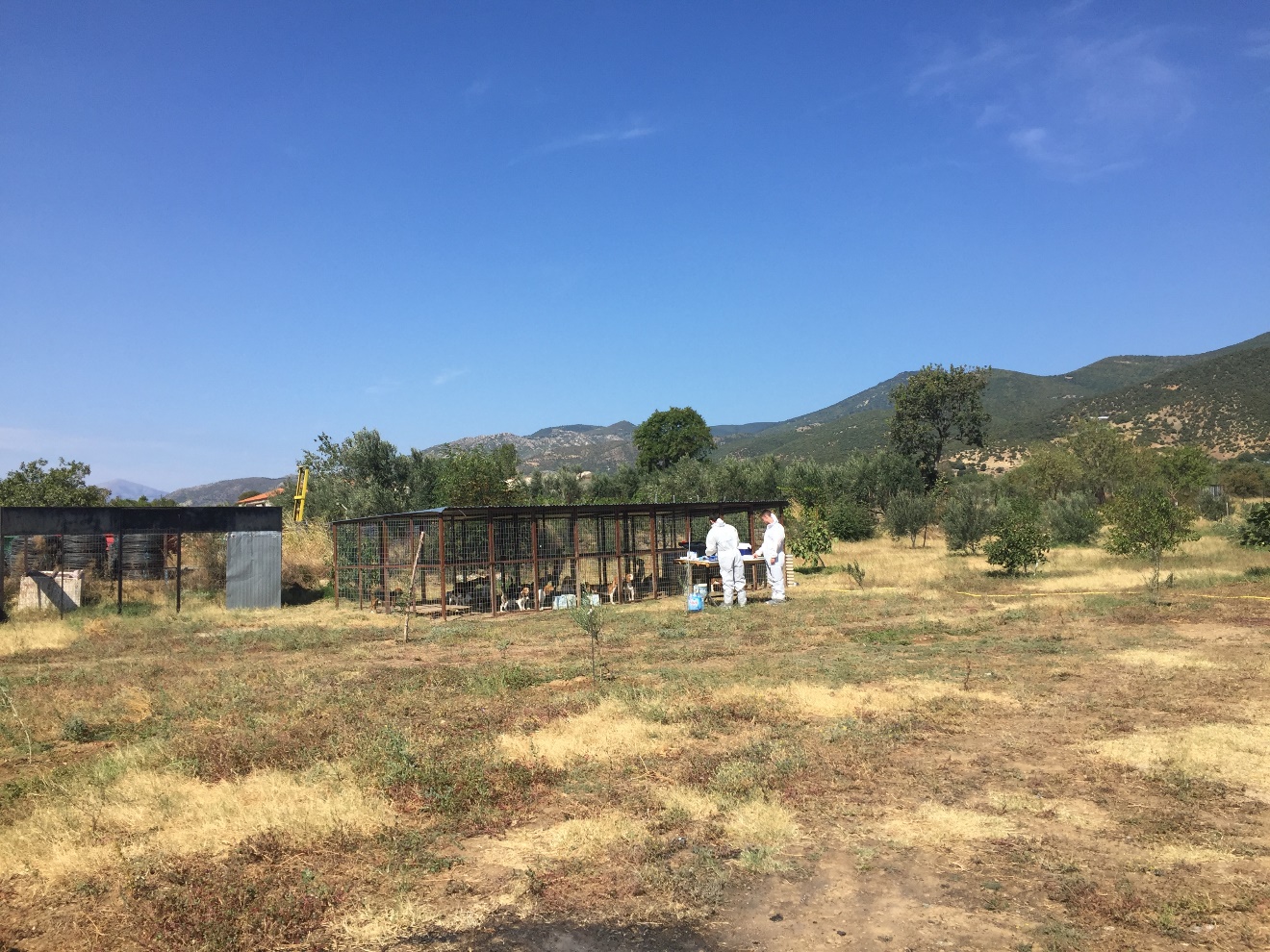


**Picture 3: Frontline Tri-Act® treated Group 2 – subunit kennel**


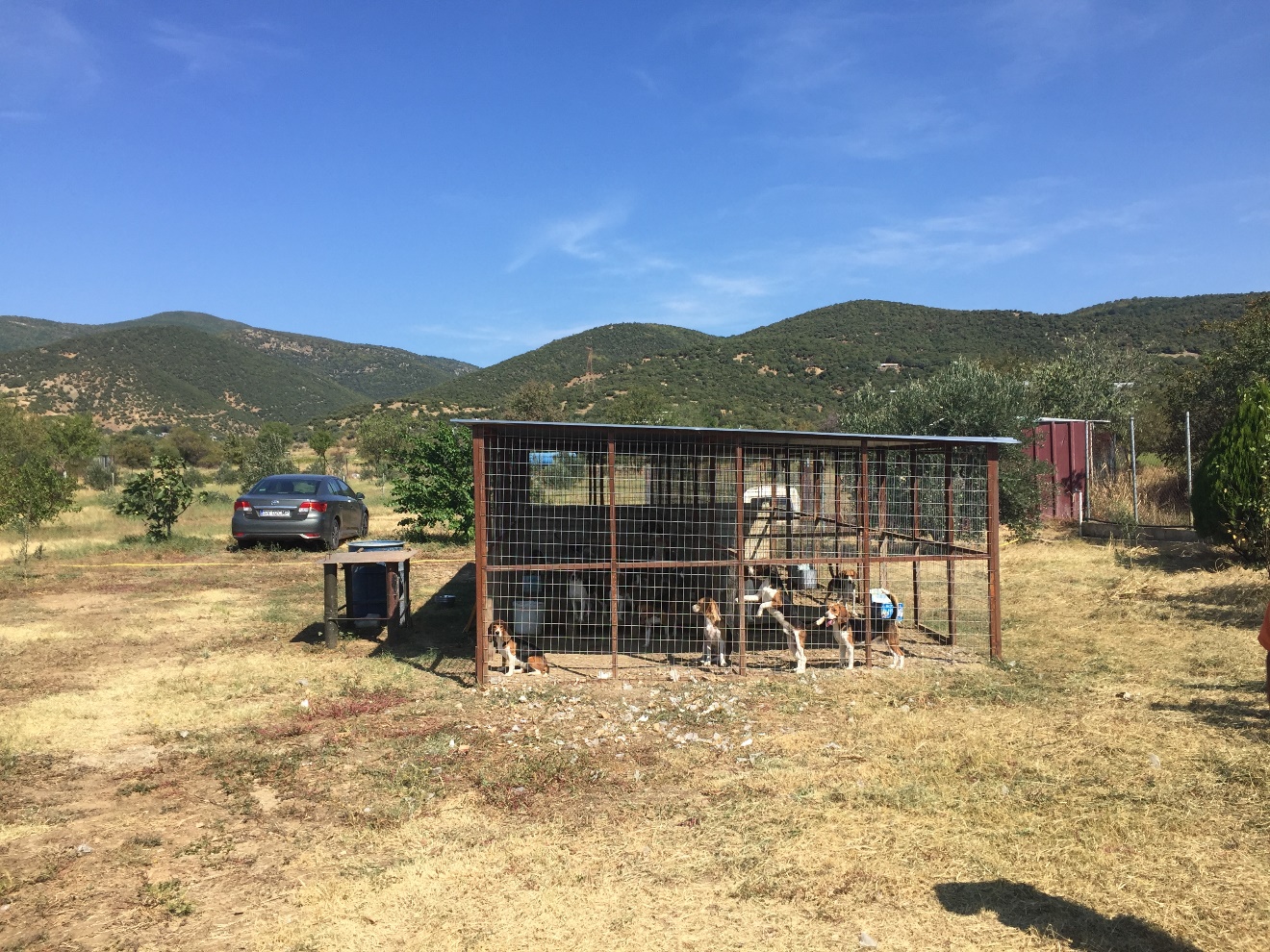


**Picture 4: Sandfly trap**


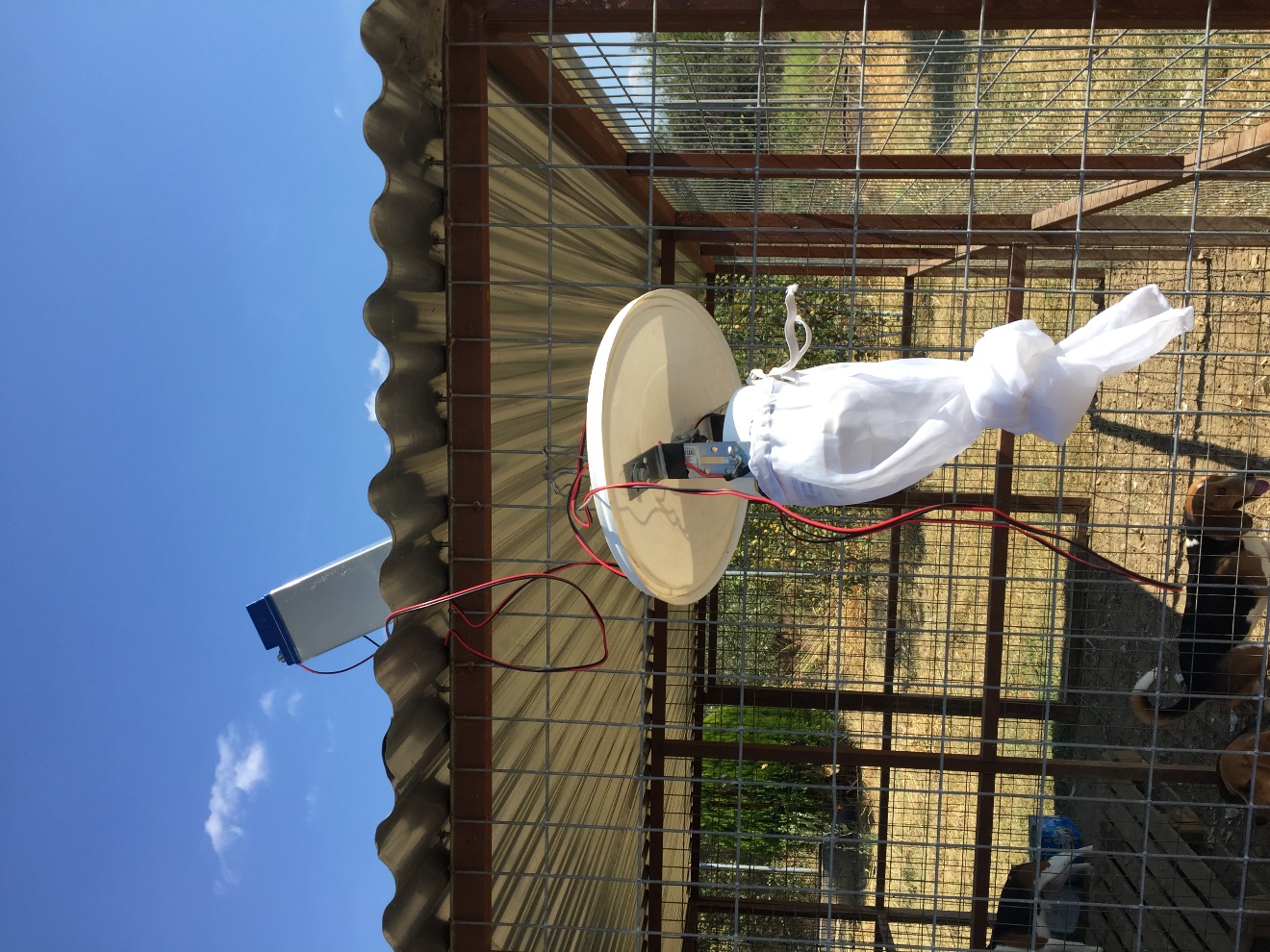


**Picture 5: Covered kennels for Phase 2**


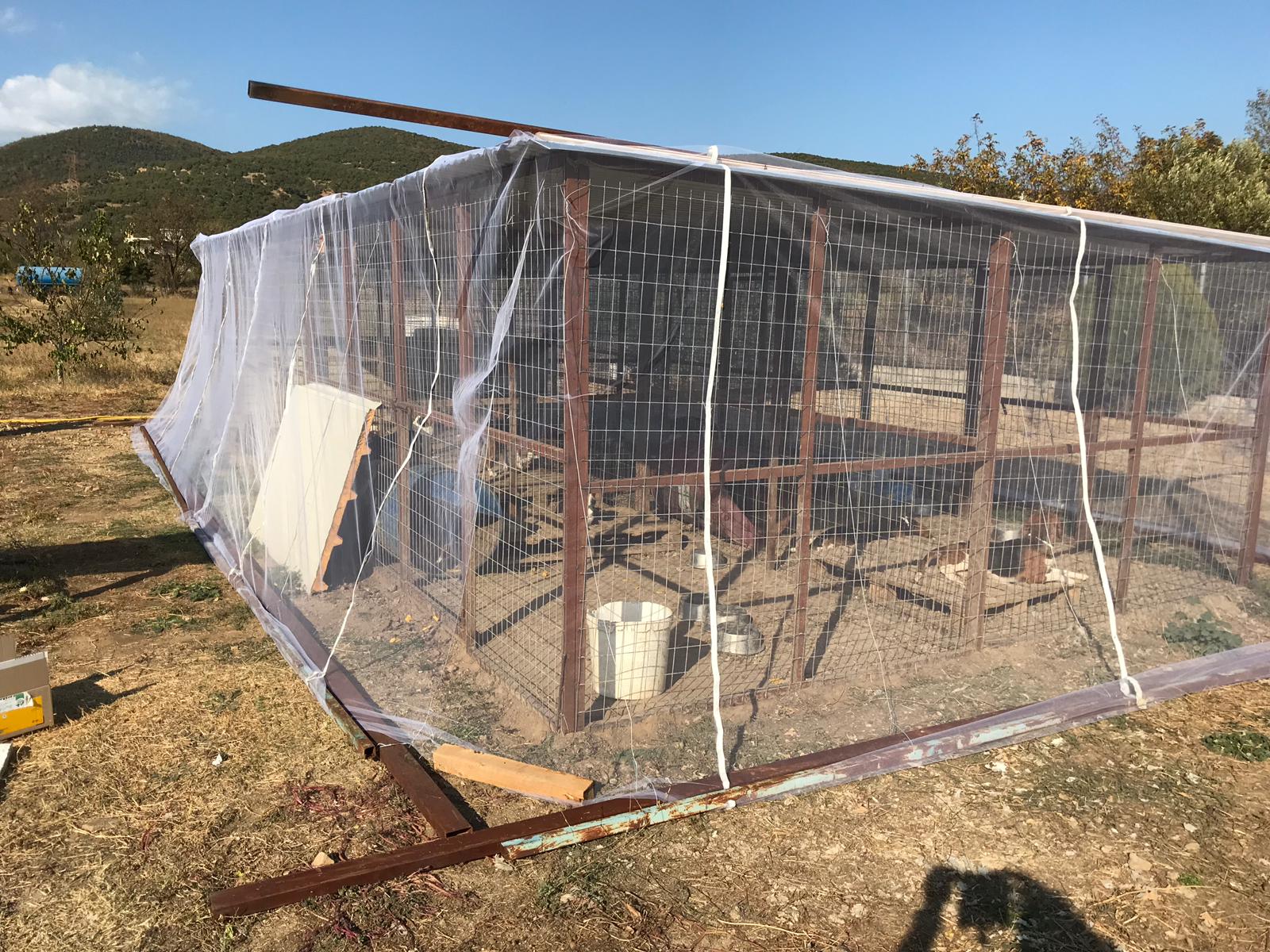

Supplement: Supplementary file 2 [file mmc2.docx]
